# Return to sports/activity level after 360° thoracolumbar fusion after burst fractures in young patients

**DOI:** 10.1016/j.bas.2024.102762

**Published:** 2024-01-23

**Authors:** Fabian Cedric Aregger, Sebastian Kreuzer, Sonja Häckel, Sebastian Frederick Bigdon, Christian Tinner, Georg Erbach, Moritz Caspar Deml, Christoph Emanuel Albers

**Affiliations:** a Department of Orthopaedic Surgery and Traumatology, Inselspital, University Hospital Bern, University Bern, Bern, Switzerland; b Department of Orthopaedic Surgery and Traumatology, Spital Thun, Thun, Switzerland; c Graduate School for Health Sciences, University of Bern, Switzerland

**Keywords:** Spinal injuries, Burst fractures, Return to work, Return to sports, 360° fusion

## Abstract

**Introduction:**

Traumatic thoracolumbar burst fractures are the most common spinal injuries and the proper treatment is controversial. In central Europe in particular, these fractures are often treated with minimally invasive anterior-posterior reduction and fusion, whereas a conservative approach is preferred in the USA. Independent of the treatment strategy, no data exists regarding the outcome related to return to activity level/sport.

**Research question:**

The aim of this study was to evaluate the return to sports and activity levels after 360° fusion in patients with thoracolumbar burst fractures without neurological deficits.

**Methods:**

Between January 2013 and December 2022, 46 patients aged 18 to 40 years underwent partial or complete vertebral body replacement in the thoracolumbar region due to traumatic burst fractures without neurologic deficit as an isolated injury. Patients were contacted retrospectively by phone calls to assess their activities using a modified version of the Tegner activity scale at different time points: Before trauma, 3, 6, and 12 months post-surgery.

**Results:**

After applying exclusion criteria, data collection was complete for 28 patients. The median modified Tegner activity scale was 5.4 before sustaining the fracture, declined to 2.9 at three months post-trauma, improved to 4.2 at six months, and reached 5.0 at 12 months. The majority (83%) of patients achieved their pre-accident activity level within 12 months. No significant differences were observed between patients with partial or complete corpectomy.

**Conclusion:**

This is the first study assessing return to sports/physical activity based on the modified Tegner scale in young patients undergoing 360° fusion for spinal burst fractures. The majority of patients (83%) return to the pre-injury activity level within 12 months after surgery.

## Highlights

• First study to use a modified Tegner scale to evaluate the activity level following thoracolumbar 360-degree fusion.
• Younger patients who underwent 360-degree fusion for thoracolumbar burst fractures demonstrated a high likelihood (83%) of regaining their original activity level.
• Compared to ACL reconstruction, these patients had a threefold higher return rate to their pre-injury activity level.

## 1 Introduction

Spinal fractures are common injuries with increasing incidence, particularly in high-income countries and the thoracolumbar region is particularly vulnerable accounting for two thirds of all spinal fractures (He et al., 2017). Men and women are affected nearly equally. Men are more frequently affected at a younger age while women experience these injuries at a later age due to underlying osteoporosis (Bigdon et al., 2022a). The most common mechanism of spinal injuries include high-energy trauma (e.g., traffic accidents, or falls from great heights, and sports) (Bigdon et al., 2022b). These injuries are significant life events for those affected and represent a relevant cost factor for healthcare and society (Wood et al., 2014; Leslie et al., 2013).

To date, there is no international consensus for the treatment of thoracolumbar burst fractures, conservative and surgical treatment modalities are applied to treat these fractures with comparable outcomes in both strategies (Pehlivanoglu et al., 2020). For the operative treatment, several concepts and combinations of anterior and posterior long or short stabilization constructs exist (Erichsen et al., 2020; Knop et al., 2009; Reinhold et al., 2010). Some advantages of anterior column reconstruction compared to isolated posterior stabilization are shown (Smits et al., 2017). The preferred technique at our institution is anteroposterior fusion (360° fusion) consisting of short segment minimally invasive posterior fusion followed by anterior column reconstruction with partial or complete corpectomy. This treatment aims to restore and maintain sagittal and coronal alignment with the shortest possible fusion distance (Smits et al., 2017). Furthermore, early surgical therapy aims to minimize the short-term impact on the social and professional life allowing early integration into everyday activities (Kerwin et al., 2007). The most frequent outcome measures in the current literature include health-related quality of life (HrQoL) and return to work (Haraldstad et al., 2019). While multiple studies have examined sport-related outcomes after knee or shoulder injuries (Giberson-Chen et al., 2023; AlSomali et al., 2021; Rudisill et al., 2021; Giesche et al., 2020; Pitsilos et al., 2022), there are only few studies assessing this aspect after injuries of the spine. Reinke et al. investigated the activity level using a modified Tegner Scale for patients after the implantation of a cervical disc prosthesis (Reinke et al., 2017). To our knowledge, no similar studies have been published for patients with these very common spinal injuries.

The aim of this study is to evaluate the return to sports and activity levels as assessed by the modified Tegner scale (Reinke et al., 2017) after 360° fusion in patients with thoracolumbar burst fractures without neurological deficits.

## 2 Methods

### 2.1 Setting/participants

After local institutional review board approval (Kantonale Ethikkommission Bern project-ID, 2021-02021), we identified all patients who underwent 360° fusion with partial or complete vertebral body replacement in the thoracolumbar junction (T10-L3) due to traumatic fractures between January 2013 and December 2022. We included all patients aged between 18 and 40 years, with written general consent. Patients treatment and demographic data was obtained from our hospital database at a single level 1 trauma center.

Patients with severe concurrent injuries that hindered rehabilitation or physical activity, e.g. polytraumatizied patients with ISS >15 or patients with lower limb injuries, and neurological status other than ASIA E (American Spinal Injury Association) were excluded.

### 2.2 Surgical technique

Before vertebral body replacement, bisegmental percutaneous, monoaxial instrumentation was conducted. Subsequently, anterior column reconstruction was performed by thoracotomy or lumbotomy depending on the fracture level. In incomplete burst fractures (Type A3 according to AOSpine Classification System) partial corpectomy removing the fractured portion of the vertebral body with the adjacent disc was conducted. Complete burst fractures (Type A4 according to AOSpine), were treated with complete corpectomy and replacement with an expandable corpectomy device. Before discharge a standing X-ray was performed.

### 2.3 Postoperative treatment algorithm

The patient was mobilized on the first day after surgery under the guidance of physiotherapy. All patients followed the same post-treatment protocol independently of disrupted or intact posterior longitudinal ligament. If a thoracic drainage was placed post thoracotomy, an attempt was made to remove it on the second day after surgery, unless contraindications were present. After mobilization, a standing X-ray was performed. Opiate-containing analgesics were ceased prior to the patient's discharge home.

After discharge, physiotherapy sessions were conducted once per week starting in the first week after the injury. In the first six weeks, the rehabilitation program included isometric training, activation, circulation promotion and movements that could increase rotational or shear force on the implanted device should be avoided. After six weeks, range of motion exercises were added. Additionally, patients were advised not to lift objects weighing heavier than 6 kg for six weeks. First follow-up visit with X-ray in two planes was performed after 6 weeks and a more progressive physiotherapy plan induced for 6 more weeks until the next follow-up visit (3 months postoperative). After pain-free completion of the physiotherapy plan patients are allowed to do sport- and occupation-specific complex high-speed strength exercises. Patients were seen for an XR control at the 6 months postoperative mark. Removal of the posterior instrumentation was indicated after 9 months at the earliest, after bony consolidation had been confirmed by CT scan (to release the caudal segment in partial corpectomies; removal in vertebral body replacement only if the instrumentation irritated the patient). If no complications occurred, final follow up was conducted 1 year postoperatively.

### 2.4 Variables/outcome measure

**Fig. 1:**
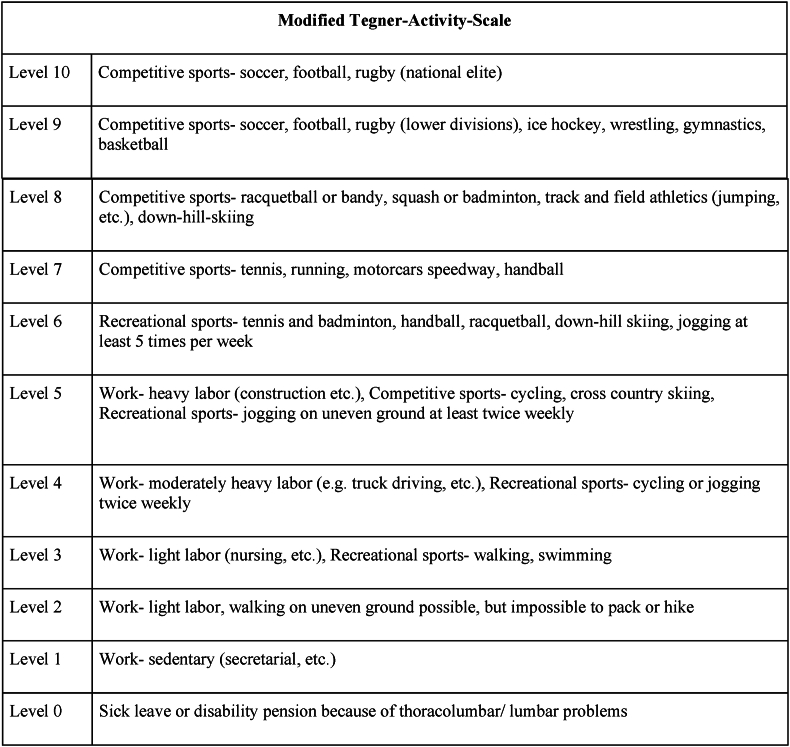
Modified Tegner Scale.

For evaluating the level of activity, we employed a previously published and (cervical) spine-adapted version of the Tegner activity scaling system^1^ (Fig. 1). The Tegner scale is graded on a ‘0-10′ format, where 10 represents international soccer-level activity and one signifies a person who is severely limited by knee issues (Tegner et al., 1985). It allows to classify the sports-related activities ranging from professional to recreational athletes to set objectives at the moment and evaluate progress. To score the thoracolumbar junction, we have only made a small adjustment from the cervical version, by adjusting the region of symptoms.

For data collection, patients were contacted through different strategies via telephone or written (mail and e-mail) (von Allmen et al., 2019). The level of activity was assessed retrospectively using the modified Tegner scale at different time points: before the trauma and 3, 6 and 12 months after the surgical procedure. Time of incapacity for work was also registered.

### 2.5 Statistical analysis

Statistical analyses were conducted with SPSS, version 28.0.1.1 (IBM Corp.) Continuous variables are presented as medians and ranges, categorical variables as frequencies and percentages. Exact Wilcoxon Signed-Rank Tests were used to compare continuous values before and after surgery. Continuous and non-continuous variables were compared using the Fischer's exact test. *P* values are two-sided, with a significance level of 0.05.

## 3 Results

Between January 2013 and December 2022, a total of 167 patients underwent partial (N = 32) or complete corpectomy (N = 135) for thoracolumbar burst fractures at our institution.

**Fig. 2:**
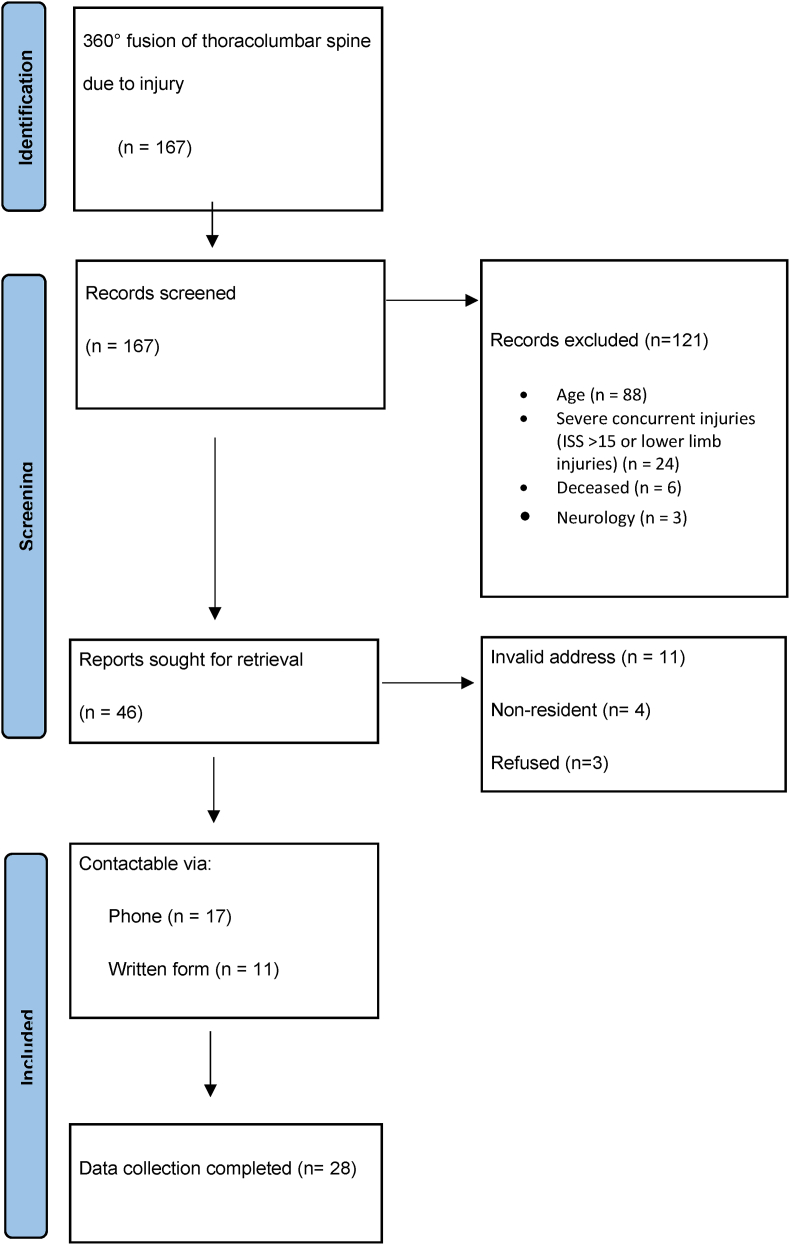
Flowchart.

Following the exclusion criteria, 46 patients remained for further analysis. Data collection was complete for 28 patients who were ultimately included in the study (Fig. 2). Of these, 19 (68%) had an injury of the posterior longitudinal ligament. In nine cases (32%) there was a compression fracture without ligament injury.

The mean age of the included patients was 26 (SD 7.4) years. The study cohort comprised 12 women and 16 men. Eleven patients (39%) underwent replacement of the complete vertebral body, while partial corpectomy was performed in 17 patients (61%). The vast majority of patients were engaged in recreational (n = 20, 71,4%) sports while only 6 (21%) were involved at a competitive level, and one at a professional level (Table 1).

**Table 1:** Patient characteristics and demographic data.

Variable	Total (n = 28)
**Female, n (%)**	12 (43)
**Age years**_**a**_**(SD)**	27 (7.4)
**Type of Sports (main activity)**	
**Running/Jogging**,**n (%)**	9 (32.5) (4x competitive, 5x recreational)
**Downhill skiing**,**n (%)**	5 (18) (1x national elite, 4x recreational)
**Hiking**,**n (%)**	5 (18)
**Soccer**,**n (%)**	2 (7) (recreational)
**Tennis**,**n (%)**	1 (3.5) (competitive)
**Volleyball**,**n (%)**	1 (3.5) (recreational)
**Cycling**,**n (%)**	2 (7) (recreational)
**Swimming**,**n (%)**	1 (3.5) (recreational)
**No sports**,**n (%)**	2 (7)
**Heavy labour**,**n (%)**	2 (7)
**Anterior surgical stabilization (%)**	
**Partial corpectomy (%)**	17 (61)
**Complete corpectomy (%)**	11 (39)

### 3.1 Analysis of activity level

For both groups, the median modified Tegner activity level was 5.4 (3-10) before the injury. At three months follow-up, the median level had declined to 2.8 (2-4), but improved at six months postoperatively, reaching a median of 4.0 (2-7). After 12 months, the median level was at 5.0 (2-10) (Table 2). One patient was professional athlete before the injury (snowboard), and returned to professional sports within 12 months.

**Table 2:** Modified Tegner Activity Scale during the 12 months follow-up.

Modified Tegner-Activity-Scale (n = 28)				
	Before Trauma	3 Months	6 Months	12 Months
**Level 10 (national league)**	1	-	-	1
**Level 9 (lower division)**	-	-	-	-
**Level 8 (racquet/ski)**	-	-	-	-
**Level 7 (tennis, running)**	6	-	2	5
**Level 6 (tennis, skiing)**	6	-	2	4
**Level 5 (jogging uneven gorund 2x/week)**	4	-	4	6
**Level 4 (cycling, jogging 2x/week)**	9	3	10	7
**Level 3 (walking, swimming)**	2	16	7	4
**Level 2 (walking uneven ground)**	-	9	3	1
**Level 1 (sedentary work)**	-	-	-	-
**Mean (SD)**	5.4 (3-10)	2.8 (2-4)	4.0 (2-7)	5.0 (2-10)

**Fig. 3:**
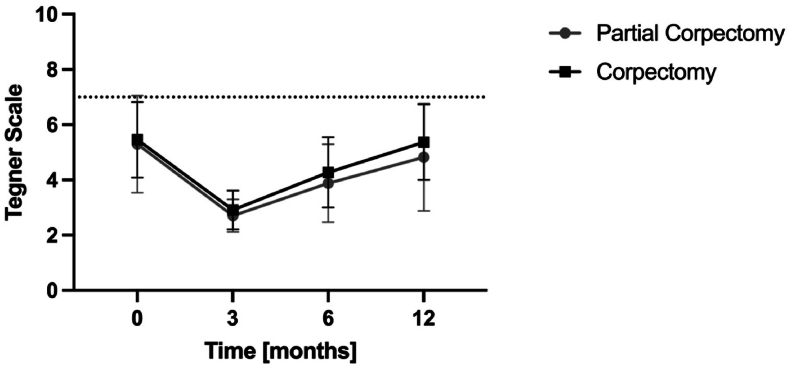
Activity Level according to Modified Tegner Activity Scale over 12 months after injury compared between partial and complete corpectomy.

No significant differences related to the activity level were observed between the partial and complete corpectomy groups (Fig. 3). Overall, 23 of 28 patients (83%) reached an activity level equivalent to their pre-injury status within 12 months (Table 2). Conversely, five patients experienced a decrease in their activity levels. Of those, one patient had a complete corpectomy and the remaining four patients partial corpectomies.

The average duration of incapacity for work for the entire study cohort was 5.8 months (2-10).

## 4 Discussion

In sport-related activities the lumbar spine experiences much higher compressive forces than in normal daily activities and work tasks (Schafer et al., 2023). To regain sports activity after a spinal injury means achievement of high capacity. Return to sports after surgical treatment of thoracolumbar burst fractures without neurologic deficit is poorly researched. No general guidelines exist but universal agreement that athletes should be neurologically intact, pain free and have full strength and range of motion before returning to play after spinal injury (Huang et al., 2016). To survey outcome parameters the AO Spine PROST (Patient Reported Outcome Spine Trauma) is common with one question to recreation and leisure but not specific on sports activity level (Buijs et al., 2022). The Tegner scale is originally and validated for the evaluation of sports level in knee injuries (Tegner et al., 1985). A modified but not validated version of the Tegner scale was used to investigate recovery after cervical spine surgery (Reinke et al., 2017).

The current study assessed the modified Tegner scale in a selected cohort of patients younger than 40 years undergoing anterior-posterior fusion of high energy thoracolumbar burst fractures. In this retrospective study, we were able to show that younger patients after 360-degree fusion for the treatment of incomplete or complete burst fractures have a high chance of regaining their original activity level (83%), irrespective of whether a partial or complete corpectomy was performed. To our knowledge, this is the first study assessing return to sports in young patients after 360° fusion for thoracolumbar burst fractures.

There is limited data to the activity level after 360° stabilization of thoracolumbar burst fractures. However, experts expect that a reemployment is possible within 4-6 months, what corresponds with our population with an average incapacity for work of 5.8 months. Schouten et al. conducted a study to determine the appropriate communication strategies that surgeons should employ when providing evidence-based information to patients regarding postoperative recovery and functional outcomes following thoracolumbar burst fractures. Fifty-one spine surgeons from Level 1 trauma centers worldwide were questioned to representative cases of thoracolumbar burst fractures and their expected outcome within one year. The questionnaires were analyzed and compared to the results of a systematic literature review. The authors summarized in their review that most of the experts expect the possibility of a return to high-level sports, whereof half expect that it will take more than 6 months and the others within 4-6 months. One third predict such an injury would end a college football career, as a sample for activity level. Twenty-nine percent predicted a return to recreational sports “without limitations", without there being reliable evidence-based information on this matter (Schouten et al., 2015). Our findings surpass the expectations published in this study. In addition, the analysis of the current study allows an estimation of which activities (based on the Tegner scale) might be possible after three (level 3), six (level 4) and 12 months (most likely aligned with the preoperative level). In the context of running, for example, this means that most individuals should be able to walk on uneven ground after 3 weeks, jog twice a week after 6 months, and run on uneven ground after 12 months.

It is known from sports-related knee injuries that only around 50% of overall patients return to their pre-injury level of sports after anterior cruciate ligament reconstruction (Czuppon et al., 2014). In recreational athletes, the rate is even lower (24%) (Kim et al., 2021). Overall, younger athletes are more likely to return to their activity levels as compared to athletes in a more advanced age (35% in <25 years old versus 12% in >25 years old) (Kim et al., 2021). The Tegner scale correlates inversely with age in patients with healthy knees, dropping remarkably after the age of 45 years (Briggs et al., 2009). Therefore, we limited the analysis in the current study to a younger population. In our cohort, we were able to show that >80% of patients return to their pre-injury activity level after a 360-degree fusion in thoracolumbar burst fractures. This is three times as high as in young recreational athletes (under 25 years of age) undergoing anterior cruciate ligament reconstruction. In our collective, 15 (53%) of the injuries were sports-related injuries. Especially in these patients, the prospect of returning to sports is an important factor, also mentally, and is very often subject of discussion in surgeon-patient talk, both preoperatively and in the follow-up visits.

### 4.1 Limitations

The assessment of activity levels relied on retrospective questioning, which may introduce recall bias as patients might not accurately remember their previous activity levels. We attempted to minimize this by matching the activity described with the consultation records, if sufficiently documented. The Tegner activity scale used in the study was modified and not originally intended and validated for evaluating spinal injuries. Although no more suitable scoring system was available, it is important to acknowledge the lack of formal validation. The study is a case series with a relatively small number of patients, which could limit the generalizability of the results. However, despite our best efforts, we were unable to contact the remaining patients. Furthermore, since 360° stabilization is our standard of care, there is no control group to alternative treatment modalities. Future studies with a prospective study design and a control group comprising conservative management as well as different surgical approaches are warranted.

## 5 Conclusion

In the current study, we could show that 83% of younger adults (<40 years old) undergoing 360° fusion for the treatment of incomplete or complete burst fractures return their initial athletic activity level one year after the injury.

## Declaration of competing interest

The authors declare that they have no known competing financial interests or personal relationships that could have appeared to influence the work reported in this paper.
